# Polarity in cuticular ridge development and insect attachment on leaf surfaces of *Schismatoglottis calyptrata* (Araceae)

**DOI:** 10.3762/bjnano.12.98

**Published:** 2021-12-01

**Authors:** Venkata A Surapaneni, Tobias Aust, Thomas Speck, Marc Thielen

**Affiliations:** 1 Plant Biomechanics Group, Botanic Garden, Faculty of Biology, University of Freiburg, Schänzlestrasse 1, 79104 Freiburg, Germany; 2 FIT, Freiburg Center for Interactive Materials and Bioinspired Technologies, Georges-Köhler-Allee 105, 79110 Freiburg, Germany; 3 FMF, Freiburg Materials Research Center, Stefan-Meier-Strasse 21, 79104 Freiburg, Germany; 4 Cluster of Excellence livMatS@ FIT- Freiburg Center for Interactive Materials and Bioinspired Technologies, University of Freiburg, Georges-Köhler-Allee 105, 79110 Freiburg, Germany

**Keywords:** cuticular ridges, insect adhesion, leaf surfaces, ontogeny, polarity, surface replication

## Abstract

The plant cuticle is a multifunctional barrier that separates the organs of the plant from the surrounding environment. Cuticular ridges are microscale wrinkle-like cuticular protrusions that occur on many flower and leaf surfaces. These microscopic ridges can help against pest insects by reducing the frictional forces experienced when they walk on the leaves and might also provide mechanical stability to the growing plant organs. Here, we have studied the development of cuticular ridges on adaxial leaf surfaces of the tropical Araceae *Schismatoglottis calyptrata*. We used polymer replicas of adaxial leaf surfaces at various ontogenetic stages to study the morphological changes occurring on the leaf surfaces. We characterized the replica surfaces by using confocal laser scanning microscopy and commercial surface analysis software. The development of cuticular ridges is polar and the ridge progression occurs basipetally with a specific inclination to the midrib on *Schismatoglottis calyptrata* leaves. Using Colorado potato beetles as model species, we performed traction experiments on freshly unrolled and adult leaves and found low walking frictional forces of insects on both of these surfaces. The changes in the micro- and macroscale morphology of the leaves should improve our understanding of the way that plants defend themselves against insect herbivores.

## Introduction

The plant cuticle is a thin non-cellular membrane that covers most of the above-ground organs of land plants. It is a composite matrix consisting of cutin and cutan as its main components, contains intracuticular waxes, and typically is covered by an outer layer of epicuticular waxes. The cuticle and the underlying epidermal cell wall are linked by a transition region that is rich in cellulose, hemicellulose, and pectin [1–5]. The outer peripheral layer of the cuticle may show various microscopic morphological structures such as cuticular ridges, epicuticular wax crystals, trichomes, and hairy structures [4,6]. The cuticular structures together with the epidermal cell shape and the cuticle chemistry provide the leaf surface with multiple functions [7]. In particular, cuticular ridges on some leaf surfaces have been found to reduce the frictional forces of insects during walking and may increase the hydrophobicity of the leaf surfaces [8–9]. On petals, they might act additionally as diffraction gratings producing structural colors to attract pollinators [10–12]. Ridges are relatively robust compared with other cuticular morphologies [8] such as epicuticular wax crystals. They may also provide mechanical stability to the growing organs by possibly avoiding cuticle cracking and maintaining structural integrity during rapid leaf area expansion [13–14].

Recently, a study of 75 different eudicot species has shown that their leaf growth is polar (or direction-dependent) and diverse and has identified four characteristic growth patterns: (1) basipetal, (2) acropetal, (3) diffused, and (4) bidirectional [15]. Such polarity in leaf growth is a result of direction-dependent cell differentiation, maturation, and proliferation processes along or perpendicular to the direction of the midrib [15–21]. One aspect that would be interesting to know is whether cuticular structures such as ridges also display such polarity during growth in the proximodistal or mediolateral axes. This is the focus of the present study. Such knowledge will help in understanding the mechanical basis of morphological changes and structural integrity of plant surfaces. Whereas the architecture of the cuticular structures and the associated functionalities have been well studied, detailed analyses concerning growth-induced changes in the morphology of cuticular structures are largely absent. Hong et al. [22] have reported that the cuticular ridges on *Arabidopsis thaliana* sepals develop basipetally, and that the ridge progression coincides with the growth and maturation of epidermal cells. Recently, Surapaneni et al. [23] have reported that, on the leaves of *Hevea brasiliensis* trees, the cuticular ridges also have a polarity during development but are characterized by an acropetally directed progression. They have also found that the ridge development coincided with the directional color changes occurring on the leaf surfaces.

Our preliminary microscopic examination and visual observations have shown that such directional color changes also occur on the cuticular ridge containing adaxial leaf surfaces of *Schismatoglottis calyptrata* (Roxb.) Zoll. & Moritzi*, Ailanthus altissima* (Mill.) Swingle and *Aesculus parviflora* Walter*.* In *S. calyptrata,* the leaves are rolled in leaf sheaths during bud formation and unroll during maturation, and thus, it is a worthwhile plant model to understand the development of cuticular ridges on the leaf surfaces with such varying macromorphology. Therefore, we have chosen *S. calyptrata* as a model plant in this study and tested the presence of polarity in the ridge development on the adaxial leaf surfaces*.* In addition, we have also tested the walking frictional forces of insects on freshly unrolled and adult adaxial leaf surfaces. *S. calyptrata* (Araceae) is a monocotyledonous species that has variegated leaves and that is distributed from Southwest China to Vanuatu [24–25]. It grows typically in tropical forest understories, forms stoloniferous (horizontal stems close to the soil surface) colonies, and can grow up to 60 cm tall. They are usually 15–50 cm long, variegated with grey-green or yellowish green spots, and form up to six leaves per crown [25–26]. In this study, we have used polymer replicas of the *S. calyptrata* leaf surfaces at various ontogenetic stages for morphometric analyses. Polymer replication of the leaf surfaces helps to avoid artifacts arising from leaf dehydration during time-consuming microscopical analyses, especially with regard to the imaging of young leaf surfaces [7,23]. By means of confocal microscopy experiments, we demonstrate that polarity in ridge development also occurs on leaves of *S. calyptrata* and that the surface roughness of the leaves increases as the leaves mature*.* Previous studies have found reduced insect adhesive forces on rough plant surfaces [8–9,23,27–31]. By performing traction experiments using Colorado potato beetles (*Leptinotarsa decemlineata*) as model insect species, we show that the walking frictional forces of insects are reduced as well on freshly unrolled as on adult leaf surfaces.

## Results

### Leaf ontogeny and replication

Leaves were in the rolled state at stage 1 (5 days from bud formation) and stage 2 (10 days from bud formation). After exponential leaf growth (Supporting Information File 1, Figure S1), the leaves unrolled at stage 3, after 12–26 days from bud appearance. Stage 4 occurred at a leaf age of 22–35 days, when the growth of the leaves as measured from the length of the midrib ceased. The leaf sizes (the leaf sheath to tip length in stages 1 and 2; midrib lengths in stages 3 and 4) varied from 0.5–5 cm at stage 1, 7–10 cm at stage 2, 11–21 cm at stage 3, to 15–24 cm at stage 4.

The polydimethylsiloxane (PDMS, positive mold) replicas of the leaf surfaces aided the study of the ontogenetic variation in the morphology of the ridges. Some leaf material, but only at stages 2A and 3, remained attached to the epoxy (negative mold) replicas during the replication process. However, in contrast to the results from Surapaneni et al. [23] on *H. brasiliensis* leaves, potassium hydroxide (KOH) treatment removed all the plant material from the epoxy surfaces, and thus, clean PDMS replicas from all leaf stages could be obtained.

### Structure of the cuticle – temporal and spatial changes

**Figure 1:**
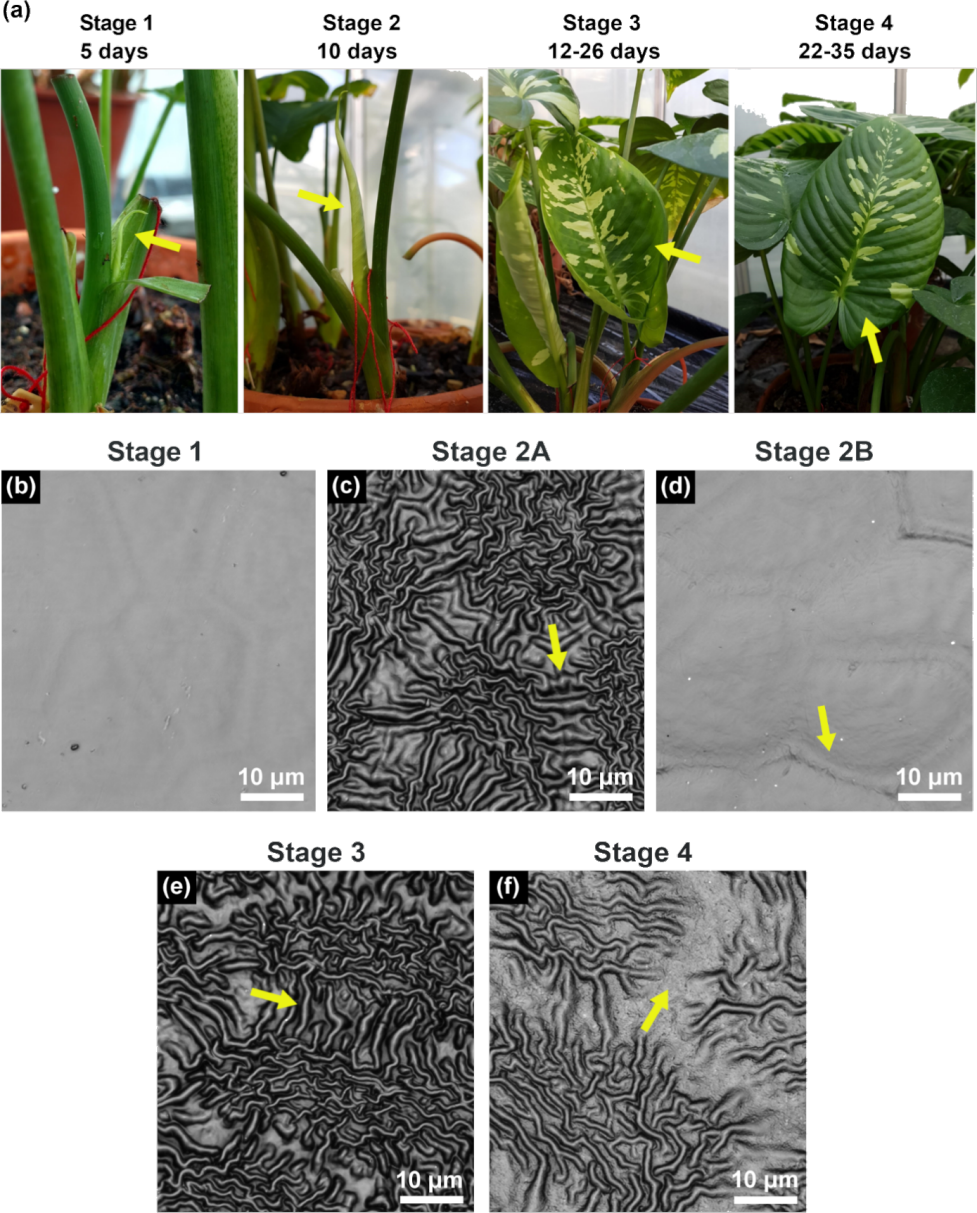
Leaf ontogeny and cuticular ridge development. (a) Leaf development from bud appearance during the various ontogenetic stages in *Schismatoglottis calyptrata.* (b–f) CLSM images of leaf replica surfaces during the different ontogenetic stages. Leaf surfaces contained smooth cells at stage 1 and stage 2B, high aspect ratio zig-zag shaped ridges at stages 2A and 3, and less dense and low aspect ratio ridges at stage 4. Arrows indicate: (a) leaves at the respective stages; (c) and (e) thick and long ridges connecting the peripheries of the adjacent cells; (d) a ridged structure along the anticlinal field; (f) the anticlinal field free of ridges.

**Figure 2:**
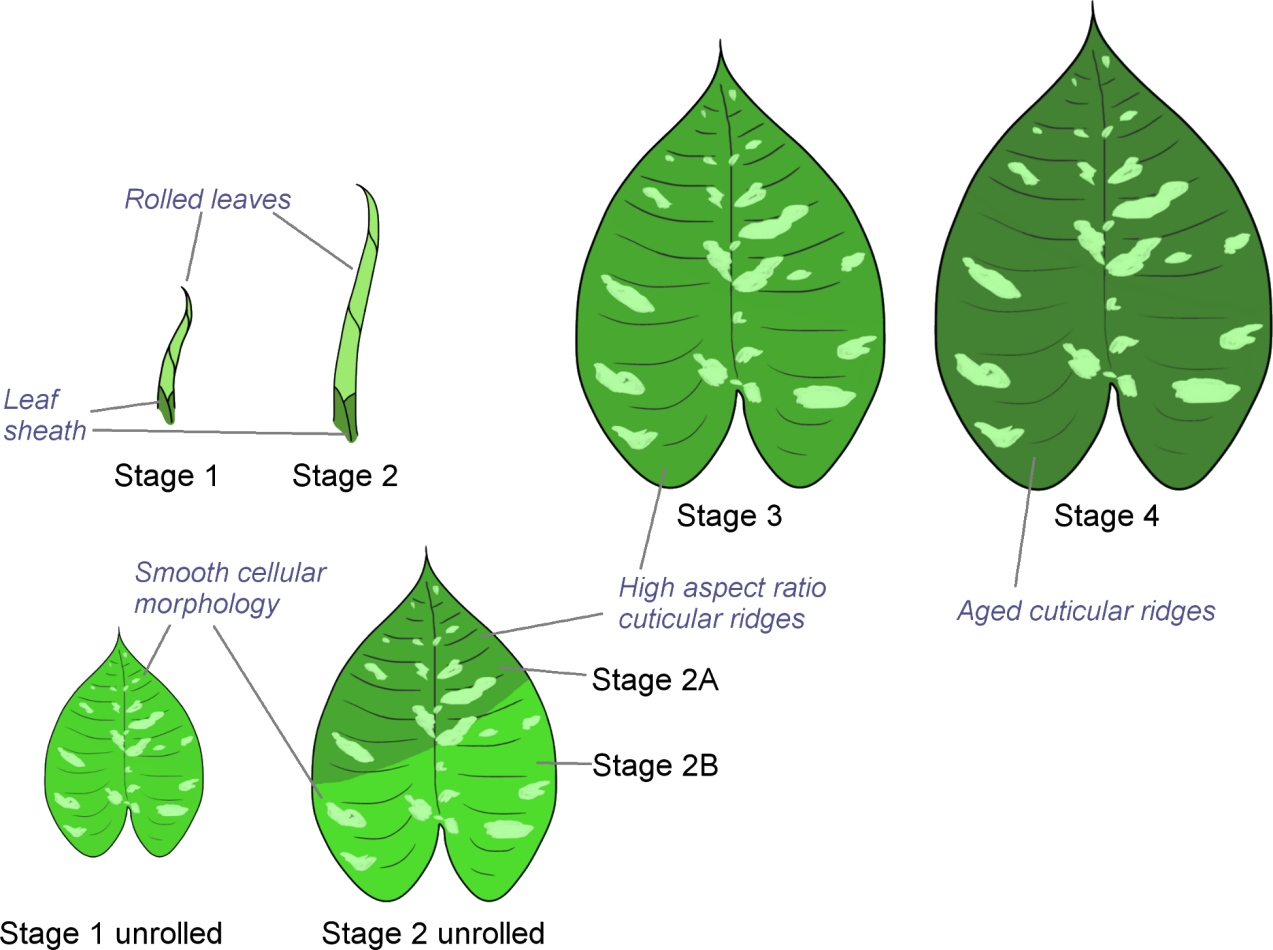
Schematic representation of leaf growth stages of *Schismatoglottis calyptrata* and their respective surface microstructures. At stages 1 and 2, leaves are normally in the rolled position. The schematics show the leaf colors when the leaves were unrolled manually.

**Figure 3:**
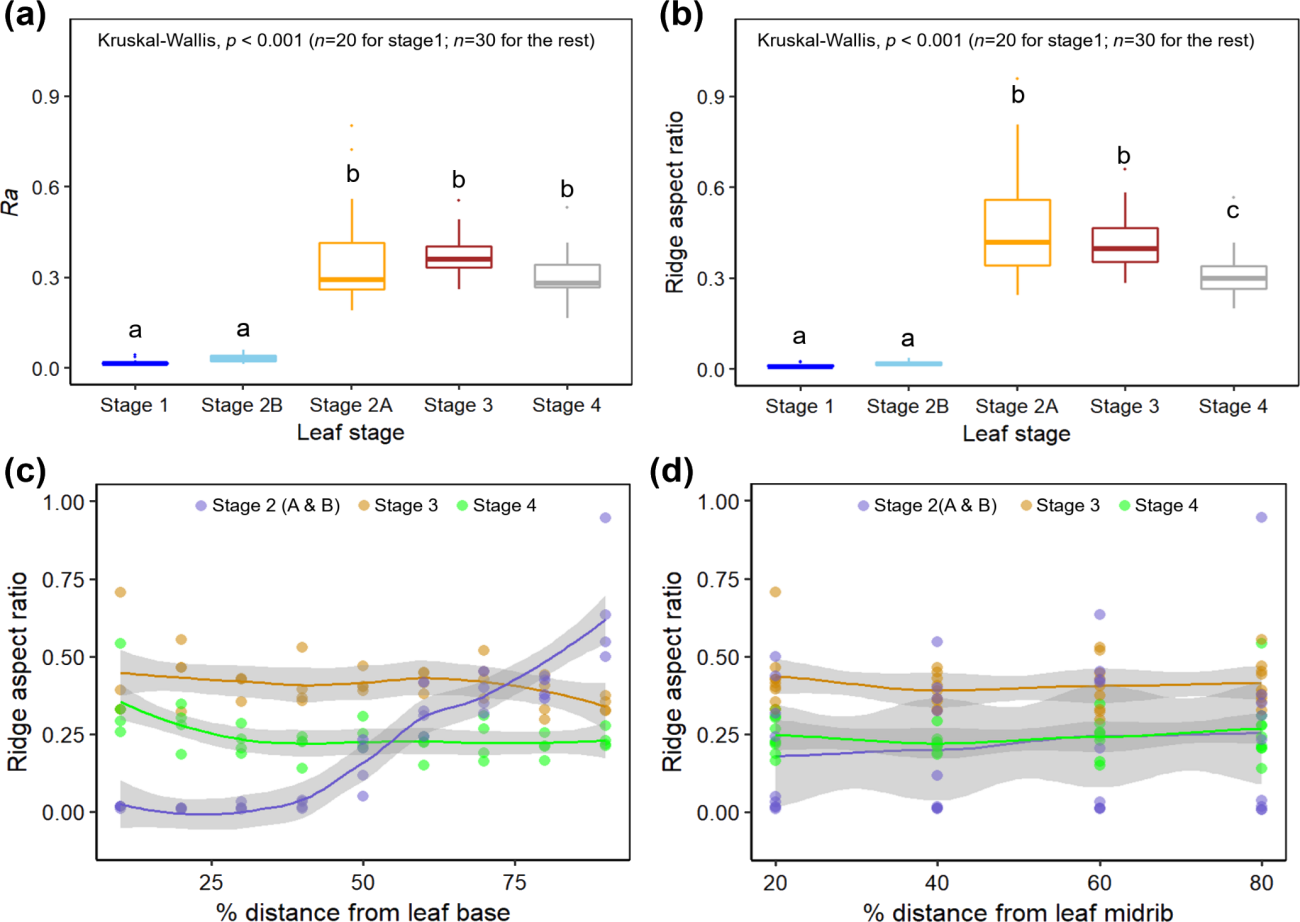
The plots show (a) the variation in the arithmetic average roughness (*Ra*) of the leaf surfaces with different growth stages, (b) the variation in the ridge aspect ratio with different leaf growth stages, (c) the spatial variation of the ridge aspect ratio along the direction of the midrib as percentage distance from the base of the leaf, and (d) the variation of ridge aspect ratio perpendicular to the direction of the midrib as percentage distance from the midrib. The gray shading around the lines represents the 95% confidence interval.

Figure 1 shows *S. calyptrata* leaves at their different ontogenetic stages (Figure 1a) and the corresponding confocal laser scanning microscopy (CLSM) observations (Figure 1b–f) on leaf microstructures. A schematic representation of the leaves and the corresponding locations of smooth and ridged morphologies is provided in Figure 2. The ontogenetic variations in roughness on the *S. calyptrata* leaf surfaces are given as the arithmetic average roughness (*Ra*) versus leaf stage in Figure 3a and as the ridge aspect ratio (*AR*) versus leaf stage in Figure 3b.

The roughness values of the leaf replicas revealed significant differences within leaf stages (Kruskall–Wallis, χ^2^ for *Ra =* 103.24, χ^2^ for *AR =* 109.24, df *=* 4, *p <* 0.001, *n*, S1 *=* 20, *n*, S2–S4 *=* 30). Figure 1b and Figure 1d demonstrate leaf surfaces with smooth epidermal cells without any cuticular structuring at stages 1 and 2B, respectively. The presence of a ridge-like structure along some anticlinal fields on the surfaces of the leaves at stage 2B (arrow in Figure 1d) was found. However, the *Ra* and *AR* of the ridges did not change significantly from stage 1 to stage 2B (*Ra*: *p* = 1, *AR*: *p* = 1, *n*, S1 = 20, *n*, S2B = 30). A basipetal progression (apex to base) of the cuticular ridges occurred with the growth of the leaves during stage 2. The morphometric analysis revealed the rapid formation of high aspect ratio cuticular ridges in the region close to the apex (stage 2A, Figure 1c). Accordingly, the *Ra* and *AR* values increased significantly at stage 2A when compared with stage 1 and stage 2B (*Ra* and *AR*: *P <* 0.001, *n* = 30). At stage 3, the entire surface of the leaves was covered with cuticular ridges as shown in Figure 1e, and no significant difference was observed in *Ra* and *AR* when compared with those of stage 2A (*Ra*: *P* = 1, *AR*: *P* = 1, *n* = 30). At stages 2A and 3, additionally, the anticlinal fields were characterized by thick and long ridges that connected the ridge islands on the adjacent cells (arrows in Figure 1c,e). As leaf growth progresses, the cuticular ridges have been reported to disappear on leaf or sepal surfaces [22,32]. However, cuticular ridges were still present on *S. calyptrata* leaf surfaces even at stage 4. Nevertheless, they were characterized by reduced height and increased spacing between the ridges (see Table 1), probably caused by cell growth (Figure 1f). The AR value decreased, but the *Ra* value did not vary significantly when compared with the values at stage 3 (*Ra*: *p* = 0.26, *AR* = 0.04, *n* = 30). A comparison of the CLSM recordings of leaf surfaces at all stages (Figure 1b–f) showed that the cells grew unevenly when they reached adult stages. Therefore, with the uneven growth of the cells, the ridges elongated (reduced zig-zag pattern), and the anticlinal fields between the cells became mostly free of cuticular ridges (arrows in Figure 1f). We observed no notable differences in the ridge dimensions between the dark green regions and the yellowish green regions on the variegated leaf surfaces (average of three spots in the dark green region: *Rc* = 0.76 µm, *Rsm* = 3.0 µm, *AR* = 0.25; in the yellowish green region: *Rc* = 0.88 µm, *Rsm* = 3.03 µm, *AR* = 0.29).

**Table 1:** Mean values of roughness parameters of cuticular ridges on leaf surfaces of *Schismatoglottis calyptrata* at stages 2A, 3, and 4.

Stage	*Ra* in µm	*Rc* in µm	*Rsm* in µm	*AR* = *Rc*/*Rsm*

stage 2A	0.36	1.03	2.16	0.48
stage 3	0.37	1.06	2.61	0.41
stage 4	0.30	0.88	2.90	0.31

The mean values of the roughness parameters are shown in Table 1. These values of mean height (*Rc*) and spacing (*Rsm*) of the ridges at the intermediate stages 2A and stage 3 are more than twice the values in the corresponding stages 2B and 3 (stage 2B: *Rc* = 0.36 µm, *Rsm* = 1.10 µm; stage 3: *Rc* = 0.42 µm, *Rsm* = 1.13 µm) on the leaves of the *H. brasiliensis* tree [23]. Surapaneni et al. [23] reported that KOH treatment was unsuccessful in removing plant material from the negative epoxy replicas. In the present study, the higher roughness (particularly, mean spacing) values on *S. calyptrata* leaf surfaces might have resulted in the easy of removal of plant material from the epoxy negatives.

Figure 3c and Figure 3d show the variation in the aspect ratio values in the proximodistal axis and the variation in the aspect ratio values along the mediolateral axis, respectively. The data were smoothened within stages by using the loess (local linear smoothing) method. We found an almost linear increase in the ridge aspect ratio beyond 40% from the base of leaves at stage 2. The area above 40% essentially represents stage 2A when the ridges start developing rapidly. At stage 3, the overall ridge aspect ratio reduced and showed further reduction beyond 70% from the leaf base. During stage 4, the aspect ratio continued to decrease slightly, until approximately 30% from the leaf base and remained constant thereafter. The aspect ratio was mostly constant in the direction perpendicular to the midrib for stages 2, 3, and 4 (Figure 3d).

**Figure 4:**
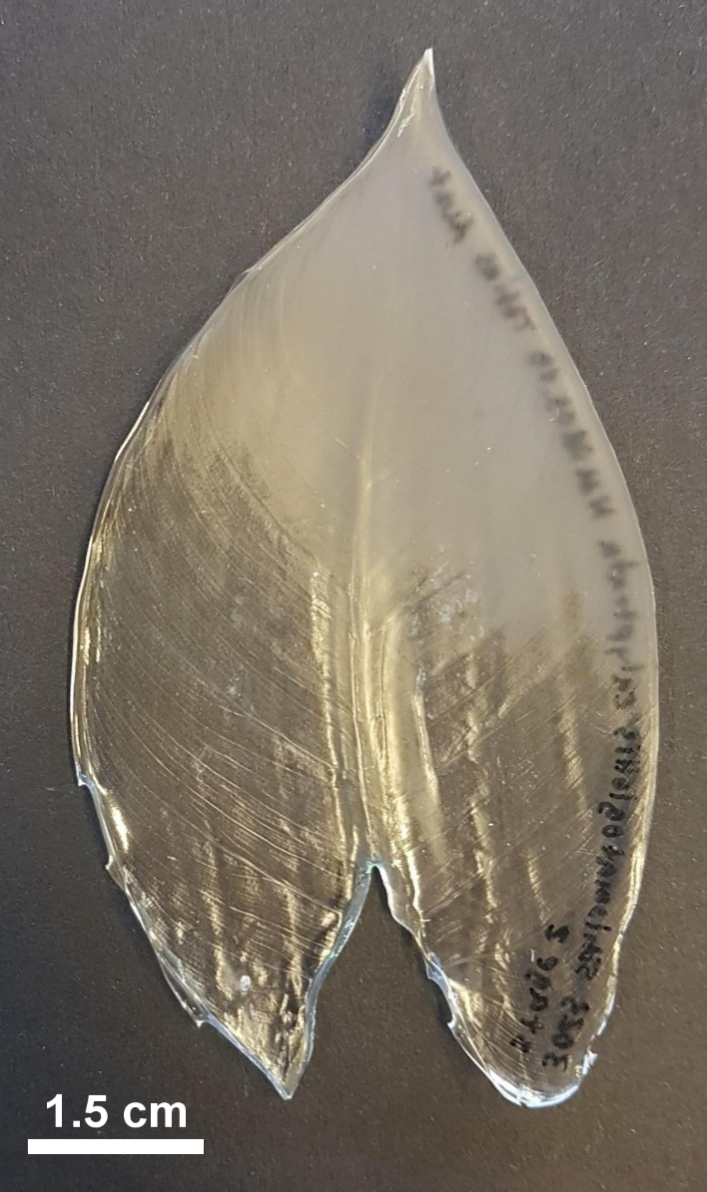
A typical epoxy replica of the surface of a leaf at growth stage 2 having a ridge progression inclined with the midrib. The transparent region represents stage 2B and contains smooth cells. The opaque region represents stage 2A and contains densely arranged high aspect ratio cuticular ridges. The inclined ridge progression might be the result of uneven exposure of the leaves to the outside environment.

The ridge progression on *S. calyptrata* leaves did not occur parallel to the proximodistal axis but at a specific inclination to the midrib (Figure 2). This could also be clearly observed from the difference in light reflection from the epoxy replicas. Figure 4 shows a typical epoxy replica of a leaf at stage 2 displaying the inclined ridge progression line. CLSM analysis showed that the surface of the transparent region of the epoxy replica corresponded to that of smooth unstructured cells, whereas the surface of the opaque region was covered with dense cuticular ridges.

**Figure 5:**
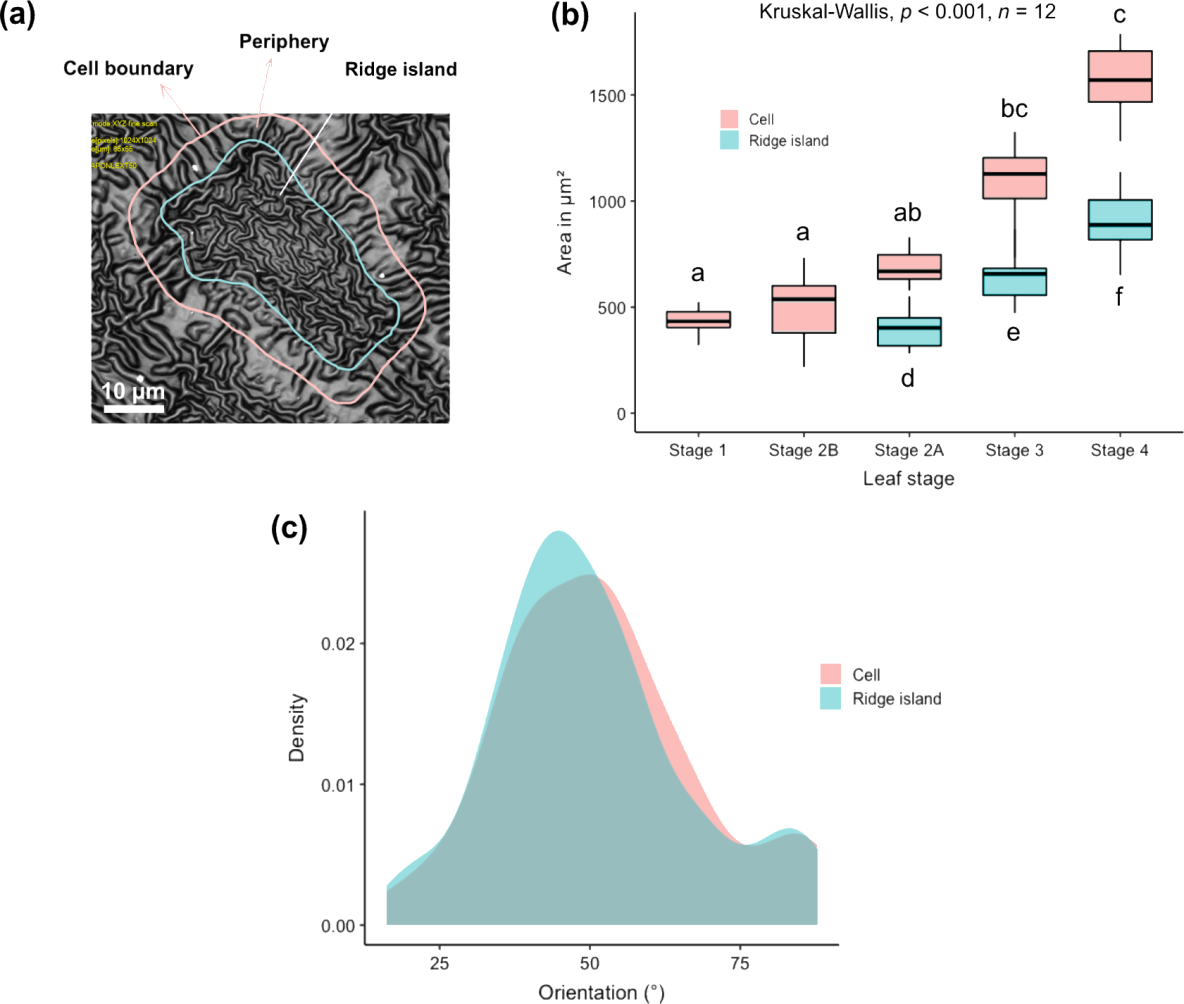
Cell size and orientation. (a) The outlines show the area of the epidermal cell as the sum of areas of ridge island, where the ridges are arranged in a nearly zig-zag pattern, and periphery, where the ridges are arranged nearly radially with respect to the center of the ridge island, (b) the boxplots show the increase in the area of cells (in pink) and ridge islands (in blue) with leaf growth, and (c) shows frequency density plots of orientations of the long axes of cells (in pink) and ridge islands (in blue) with respect to the midrib of the leaf.

Analysis of individual epidermal cells demonstrated the change of size and orientation of the cells and the ridge islands during the ontogeny (Figure 5). Figure 5a demonstrates the demarcation of ridge islands and the periphery. The variation in the area of ridge islands and the epidermal cells during ontogeny are plotted as boxplots in Figure 5b. There are significant differences in the cell size (Kruskall–Wallis, χ^2^ = 51.35, df *=* 4, *p* < 0.001, *n* = 12) and ridge island size (Kruskall–Wallis, χ^2^ = 25.18, df *=* 2, *p* < 0.001, *n* = 12) during ontogeny. Pairwise analysis of cell area showed no significant differences between adjacent stages (Figure 5b). The cell size is, however, significantly different between stages 1 and 3 (*p* < 0.001, *n* = 12), stages 1 and 4 (*p* < 0.001, *n* = 12), stages 2A and 4 (*p* < 0.01*, n* = 12), stages 2B and 3 (*p* < 0.01, *n* = 12) and stages 2B and 4 (*p* < 0.001, *n* = 12). Note that the cell size data are normally distributed and the variances are homogenous. Since the data were collected from the same leaf replica, it was assumed that the data were not independent. If the data were assumed to be independent, ANOVA analysis showed significant differences (*p* < 0.001, *n* = 12) in cell size between all the stages except between stages 1 and 2B (*p* < 0.80, *n* = 12). The size of ridge islands differes significantly between adjacent leaf stages (*p* < 0.01 between stages 2A and 3 and stages 3 and 4, and *p* < 0.001 between stages 2A and 4; *n* = 12). Figure 5b also shows that the difference between the median values of cell and ridge island areas increases with leaf growth, demonstrating the increase in the area of the periphery or the anticlinal region with cell growth. The variation in the density of cells and ridge islands (as number of cells per square millimeter and ridge islands per square millimeter, respectively) with leaf growth is shown in Table 2. The density of the ridge islands is almost twice as that of the cells in all stages of ridge progression and growth. Figure 5c shows the frequency distribution of the orientation of the long axes of cells and ridge islands with respect to the midrib of the leaf at all growth stages. Typical examples of data on the orientation of ridges in the ridge islands and the periphery regions for the different growth stages are shown in Supporting Information File 2. The analysis showed that, in the periphery region the ridges were radially oriented relative to the centre of the ridge island during all stages of ridge progression and growth. In the ridge islands, however, the ridges had a complex zig-zag-like orientation during stage 2A. Similarly, during stage 3, the ridges were mostly oriented in a zig-zag manner, although on some cells longitudinal alignment of the ridges along the long axes of the cells was observed. During stage 4, the ridge orientation was mostly longitudinally oriented along the long axes of the cells.

**Table 2:** The variation in the density of cells and ridge islands (as number of cells per square millimeter) with leaf growth.

Stage	Density of ridge islands per square millimeter (*r*)	Density of cells per square millimeter (*c*)	*r*/*c*

stage 1		2322.41	
stage 2A	2482.52	1423.08	1.74
stage 2B		2000.21	
stage 3	1609.37	892.44	1.80
stage 4	1115.81	626.60	1.78

### Insect traction forces

**Figure 6:**
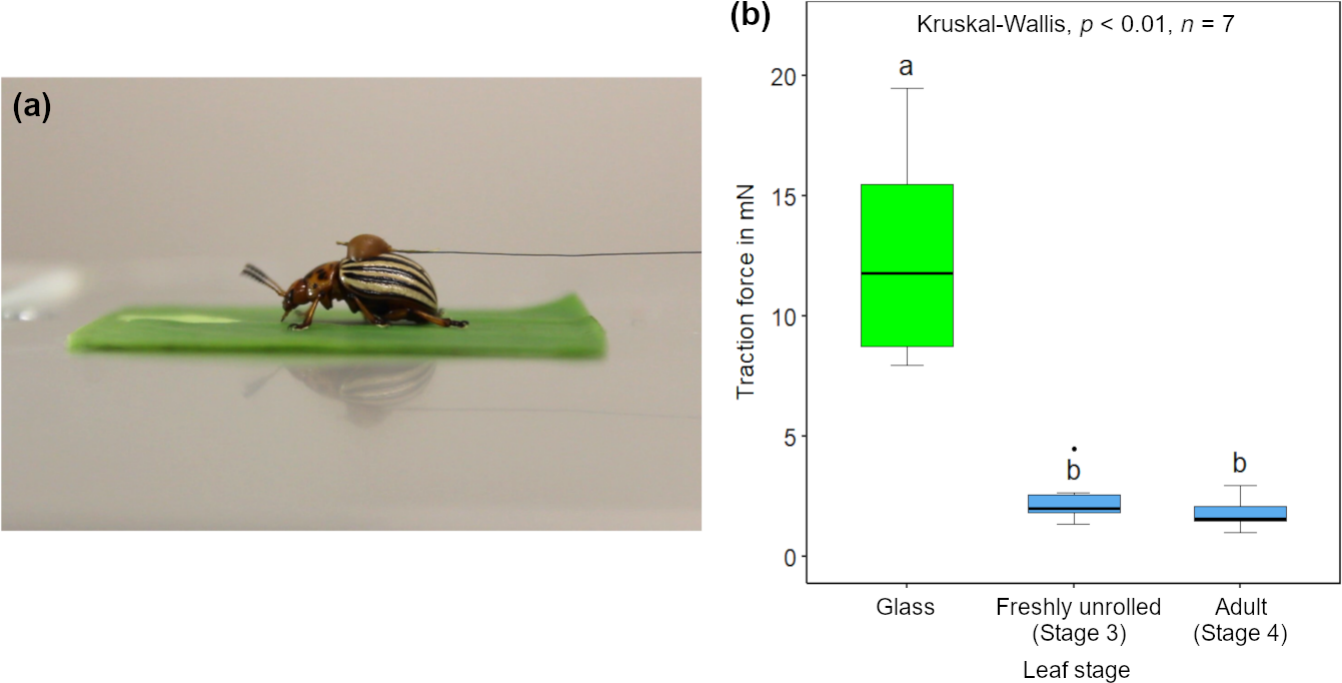
Insect traction forces: (a) A female Colorado potato beetle walking on a *Schismatoglottis calyptrata* leaf sample and (b) traction forces (in mN) of female Colorado potato beetles on glass, freshly unrolled (stage 3) and adult (stage 4) leaf samples (*n =* 7).

The maximum insect traction forces on freshly unrolled (stage 3) and adult (stage 4) *S. calyptrata* leaves differed significantly from those on glass (Kruskal–Wallis, *p* < 0.01, *n* = 7). Figure 6 shows the variation in the traction forces of insects on glass and leaf surfaces. When compared with the forces on glass (*F*_median_ = 11.79 mN), the traction forces of insects are reduced significantly on stage 3 (*F*_median_ = 2.02 mN, *p* < 0.05, *n* = 7) and stage 4 (*F*_median_ = 1.56 mN, *p* < 0.01, *n* = 7) leaves. However, the forces did not differ significantly between stage 3 and stage 4 leaves (*p* = 1.00, *n* = 7).

## Discussion

In this study, we have demonstrated that the morphology of the cuticular ridges on the adaxial side of *S. calyptrata* leaves changes significantly during leaf ontogeny. Similar to *H. brasiliensis* [23], the ontogenetic changes on *S. calyptrata* leaf surfaces revealed three distinct levels of cuticular morphology that coincided with the color changes on the leaf surfaces, albeit with few differences. Supporting Information File 1, Figure S2 shows the comparison of these distinct morphological levels on *H. brasiliensis* and *S. calyptrata* leaf surfaces. The first level is characterized by smooth epidermal cells with no superimposed cuticular structures, which occurred from the bud appearance until stage 1 in both species, and further in stage 2A (region towards apex) in *H. brasiliensis* (Supporting Information File 1, Figure S2a) and stage 2B (region towards base) in *S. calyptrata* (Supporting Information File 1, Figure S2d). The second level pertains to densely arranged high aspect ratio ridges oriented in a zig-zag manner, which developed acropetally from stages 2B and 3 in *H. brasiliensis* (Supporting Information File 1, Figure S2b) and basipetally from stages 2A and 3 in *S. calyptrata* (Supporting Information File 1, Figure S2e). In this level, the ridges on *S. calyptrata* leaf surfaces were thicker and more loosely packed (also see Results section) with larger mean spacing between the ridges when compared with those on *H. brasiliensis* leaves*.* These morphological dissimilarities of the cuticular ridges might have contributed to the differences in plant material separability from the negative molds in both species. In addition to possible chemical interactions between leaf material and epoxy during replication, the mean spacing between the ridges might be a factor enabling the efficient separation of plant material from the epoxy negative molds during polymer replication. The third level is characterized by aged and uniformly aligned low aspect ratio ridges in a labyrinth-type arrangement (at stages 4 and 5) in *H. brasiliensis* (Supporting Information File 1, Figure S2c), and low aspect ratio, thick, elongated and spaced ridges with smooth anticlinal fields (at stage 4) in *S. calyptrata* (Supporting Information File 1, Figure S2f). In this level, in *S. calyptrata* leaf surfaces, the ridges also have predominantly longitudinal orientation representing stretching of the ridges during cell expansion. From the second level to the third level, the ridge islands and the anticlinal fields undergo growth-induced expansion resulting in the observed morphological changes and alignment of ridges. Hong et al. [22] also reported that the densely arranged cuticular ridges on wild-type *Arabidopsis* sepals form at an intermediate stage with a basipetal progression and that the density of the ridges reduces as the cells mature. They have also shown that, on *Arabidopsis* sepals, the cells beneath the ridges mature at the same time as ridges form over the cells. Assuming that this is also the case in *S. calyptrata* leaves, the levels of ridge formation, development, and morphology appear to follow a general pattern based on the underlying cellular processes in both leaves and sepals.

In contrast to *H. brasiliensis* leaves and *Arabidopsis* sepals, the ridge progression on S*. calyptrata* leaves occurs with a specific inclination to the midrib. Notably, *S. calyptrata* leaves remain rolled-up from bud formation until stage 3. Moreover, when the ridges start to develop (stage 2A, in the region close to the leaf apex), the region close to the base of the leaves still lies within the leaf sheath, and the leaf margin over the rolled leaf covers the leaf in an inclined fashion (intuitive from a simple rolled paper experiment). The rolling of the leaves during the initial developmental stages might be an evolutionary trait because of various abiotic factors such as water stress, temperature, and excess radiation and/or might represent a defense mechanism against, for example, insects or viruses [33–35]. When the leaves are rolled, the intensity of light reaching each underlying layer of the rolled-up leaf and the transpiration properties within each layer will be different. Such unevenness in the exposure of the leaf to the external environment might result in the inclined ridge progression on the *S. calyptrata* leaves. Also, the presence of ridges on the leaf surfaces might reduce friction, thereby, avoiding damage between the delicate rolled leaf layers during unrolling and growth.

Previous studies on cuticular ridge development [22–23] and our observations of *S. calyptrata* leaves as presented in this study also establish that the formation and the development of cuticular ridges on plant leaf and sepal surfaces display polarity in various plant species, probably correlated with the polarity in leaf and sepal growth. Whereas cuticular ridge development is known to have polarity, we need to understand the mechanical effects required for such development. During young leaf stages, the thickness of the cuticle is usually low, and the growth of the leaves is, in general, accompanied by the thickening of the cuticle, because of the addition of cuticular material [3]. Martens [36] predicted that the overproduction of cuticle material induces the formation of cuticular ridges, a predication supported by a recent model [37]. In environments with high temperatures, low humidity, and high light intensity, plants are known to develop thicker and (more) waxy cuticles [38–40]. Therefore, as described in the previous section, the ridge development on the leaves (Figure 2 and Figure 4) might depend upon the way that leaves are covered and protected (by being rolled) during growth and their interaction with the external environment.

Mechanically, when the strain in the cuticle (induced by the simultaneous isotropic production of the cuticle and anisotropic expansion of the underlying cells) increases beyond a critical strain value, ridges start to develop [37,41–42]. Moreover, the amount of polysaccharide and cutin components present in the cuticle probably influences the stiffness and elastic behavior of the cuticle–cell wall interface [43] and certainly affects ridge formation. The thickened cuticle provides structural support to the growing epidermal cells [14], and the ridges might help in maintaining the structural integrity of the cuticle by avoiding cracking during rapid leaf expansion. The way that the amount and arrangement of the cuticular components vary during the cellular growth processes throughout ontogeny might determine the differences in cuticular morphology among the various plant species or the means by which polarity is established in cuticular (ridge) development in a given species. Vice versa, the variation in the morphology of the ridges and the underlying mechanics should enable inferences to be made regarding the underlying structure of the cuticle–cell wall interface. A better understanding of these processes might also provide insights for bioinspired growth or swelling-induced microstructures for technical applications [44].

The morphological changes in the cuticular structure of plant leaves during ontogeny have been demonstrated to influence insect attachment [23]. Our experiments showed reduced traction forces of the model insects (female *L. decemlineata*, Coleoptera) on freshly unrolled and adult *S. calyptrata* adaxial leaf surfaces. The reduction in the traction forces of the beetles was almost 83% on the freshly unrolled leaf and almost 87% on the adult leaf, when compared with those on glass. Earlier studies have found similarly large reduction in the traction forces of Colorado potato beetles on leaves with cuticular ridges and their replicas when compared with those on glass [8,23]. Therefore, from the point of leaf unrolling until adult leaf stages of *S. calyptrata,* the cuticular structure may influence the attachment and, thus, the activity of herbivores or pollinators (associated with the order, Coleoptera [45]). During young stages, however, the *S. calyptrata* leaves remain rolled-up, a type of macroscale morphology that might also reduce herbivory [34].

## Conclusion

CLSM measurements of PDMS replicas of *Schismatoglottis calyptrata* leaves have shown that the cuticular ridges display pronounced polarity and morphologically distinct levels during development. We have found smooth cellular surfaces on young leaf surfaces and ridges developing basipetally on leaf surfaces at an intermediate leaf stage. As soon as ridge development starts, the ridge aspect ratio immediately increases almost linearly on the same leaf in the direction parallel to the midrib. The aspect ratio, however, is significantly reduced on adult (aged) leaf surfaces, with the anticlinal fields being almost free of ridges. The variations in the morphology of cuticular ridges shown in the present study and from previous studies suggest a general (three-level) pattern of ridge development in various plant species and organs. We have also found that ridge progression occurs at an inclination to the proximodistal axis on *Schismatoglottis calyptrata* leaves, possibly a result of leaf rolling and nonuniform exposure to the environment during early leaf stages. Our results can be extrapolated to the mechanistic basis of cuticular structure (ridge) development and might, thus, be of interest with regard to bioinspired applications. While the macroscale morphology of the leaves is expected to influence insect attachment during young leaf stages, the cuticular microstructuring on the leaf surfaces influences insect attachment during mature leaf stages.

## Experimental

### Leaf collection and ontogenetic stages

*S. calyptrata* plants (potted plant <0.5 m height) were cultivated in the glass houses of the Botanic Garden of the University of Freiburg, Germany. The average temperature and average relative humidity in the glass houses were 22.6 ± 2.6 °C and 73.5 ± 11.0%, respectively. Voucher specimens (FB 15013) of the leaves are deposited in the herbarium of Freiburg, University of Freiburg, Germany. The leaf growth was monitored from bud to adult stages. Initial trial experiments by using a confocal laser scanning microscope helped in the classification of the growth stages, which is based on the microstructural morphology of the adaxial leaf surfaces and leaf age. In total, four ontogenetic stages were defined. Stage 1 leaves had smooth epidermal cells with no cuticular structuring. As the young *S. calyptrata* leaves were still rolled-up at this stage, the ontogenetic stage was defined based on an arbitrarily chosen leaf age, namely five days from bud formation. Stage 2 leaves had cuticular ridges covering half (50 ± 10%) of the leaf surface. This was subdivided into stage 2A representing the area toward the apex of the leaves and stage 2B representing the area toward the base of the leaves. Since stage 2 leaves also remained in a rolled-up state, we conducted initial trial experiments to estimate the age of the leaves (after 10 days from bud formation) and collected the leaves at this age. The length of the leaves at stages 1 and 2 was considered to be the length from the end of the leaf sheath to the tip of the leaf. The leaves unrolled at stage 3. At this stage, the entire surfaces were covered with cuticular ridges. Stage 4 leaves were adult leaves the growth of which (as deduced from the length of the midrib) was completed.

### Surface replication and characterization

**Figure 7:**
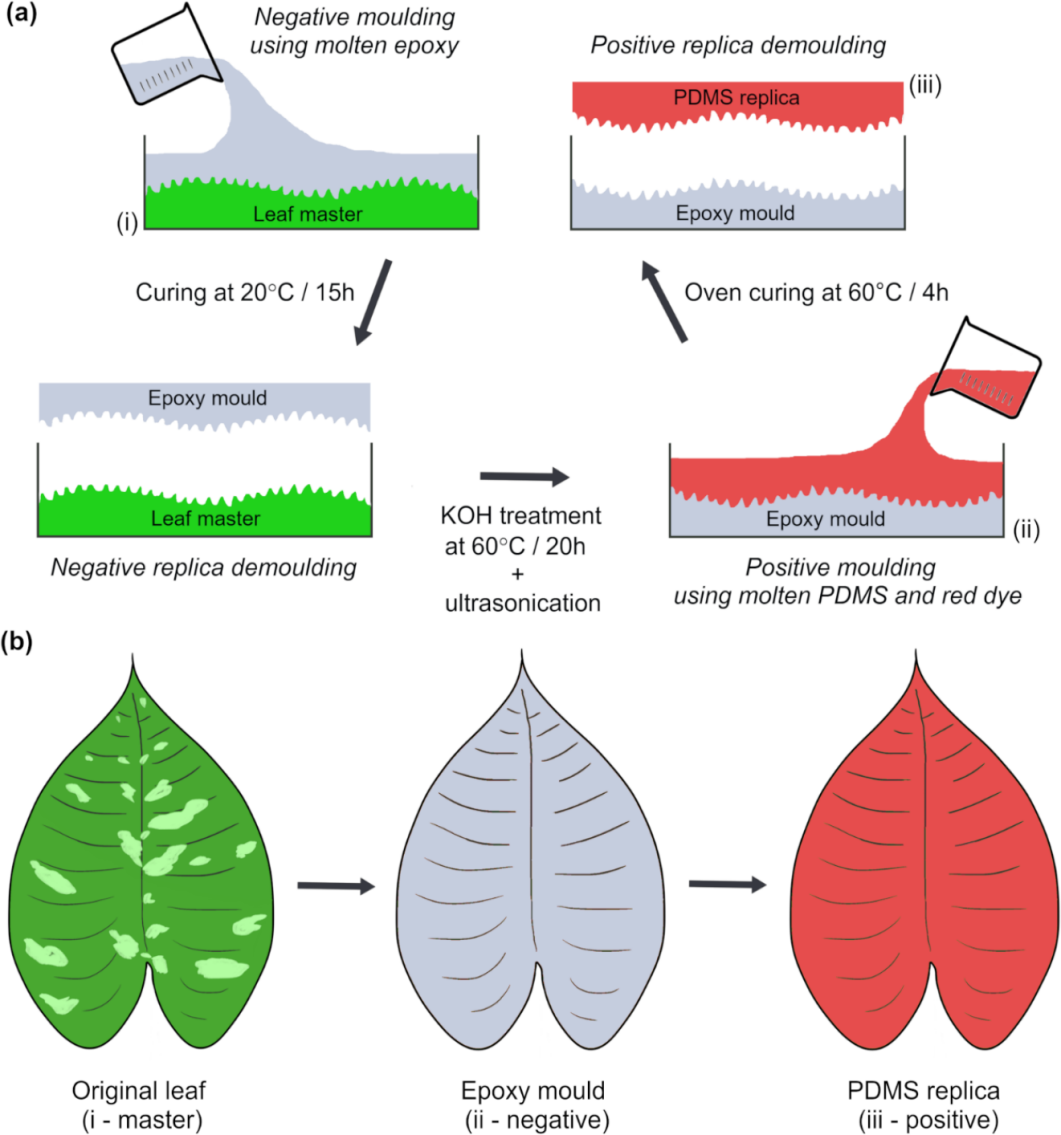
Schematic representation of (a) the replication process of *Schismatoglottis calyptrata* leaf surfaces and (b) original, epoxy negative and PDMS positive replicas. The leaves at stages 1 and 2 were unrolled for replication and the adaxial side of the leaves at all stages were replicated.

Replication of the leaf surface was carried out using a two-step molding approach (Epoxy-PDMS) as described in Kumar et al. [46] and Surapaneni et al. [23], except that the entire adaxial leaf surfaces of *S. calyptrata* (leaf area: 15–293 cm^2^) were replicated in our study. The leaves of *S. calyptrata* at stages 1 and 2 were un-rolled gently before replication, and all the leaves were attached to a clean flat plate by using double-sided adhesive tape (Tesa SE, Norderstedt, Germany) with the adaxial side of the leaves facing upward. The replication process was started within 10 min after the leaves were cut. Epoxy (Epoxy Resin L & Hardener S, Toolcraft, Conrad Electronic SE, Hirschau, Germany; resin to hardener mixing ratio of 10:4.8) negative molds were then obtained from the leaf (master) surfaces. After the epoxy molds were cured, polydimethylsiloxane (PDMS, Bluesil ESA 7250 A & B kit, Bluestar Silicones GmbH, Leverkusen, Germany; weight ratio of 10:1) positive molds dyed in red were obtained from the negative molds and allowed to cure. A detailed explanation of the replication process is given in Kumar et al. [46] and Surapaneni et al. [23], and is shown schematically in Figure 7a and Figure 7b. A total of three leaves in stages 2, 3 and 4, respectively, and two leaves in stage 1 were replicated.

The replicas of freshly cut leaves at the different ontogenetic stages were characterized using a confocal laser scanning microscope (CLSM, Olympus LEXT OLS4000, 405 nm laser, Olympus Corporation, Tokyo, Japan). To assess the statistical differences in ridge morphology on the leaf surfaces of *S. calyptrata* during ontogeny, ten different spots/areas (65 × 65 µm) on both sides of the midrib of each leaf replica were recorded. In order to study the spatial variation in ridge morphology, single leaf replicas from stages 2 (2A and 2B together), 3, and 4 were tested, and a total of 36 spots (130 × 130 µm) on the left side of the midrib of the leaves were recorded. These spots lay at the intersections of four equal divisions parallel to the midrib and nine equal divisions perpendicular to the midrib of the leaves. The distances from the midrib at each vertical division were measured to obtain the divisions in the parallel direction (Supporting Information File 1, Figure S3). Commercial surface analysis software (Mountains Map Premium version 7.4, Digital Surf SARL, Besançon, France) was used to analyze the CLSM measurements. After the filtering of median noise, zig-zag profile lines of at least 200 µm in length were taken on the topographic layer of each spot. A standard Gaussian filter (8 µm) was applied to the profiles in order to separate waviness and roughness. In order to compare the variation in the ridge morphology during growth, we calculated standard line roughness parameters, namely *Rc* (mean height), *Rsm* (mean spacing), *Ra* (arithmetic mean height) [47], and the aspect ratio of the ridges defined as *AR* = *Rc*/*Rsm*.

In addition, we selected single epidermal cells within the spots, and on each cell, we differentiated between “ridge island” as the central region with zig-zag or longitudinal arrangement of ridges (i.e., ridges not radially arranged) and “periphery” as the transition zone between the ridge island and the anticlinal field, featuring ridges that are radially oriented with respect to the cell center. The center of the anticlinal field was used to demarcate the adjacent cells. A total of twelve epidermal cells were analysed within each ontogenetic stage. We used ImageJ software (FIJI, version 1.53f51) to assess the ontogenetic differences in size and orientation of the ridge islands and the epidermal cells (ridge island + periphery, also see Figure 5a). The directionality plugin (version 2.3.0) in ImageJ software was used to analyse the orientation of ridges in the ridge island and the periphery at different growth stages.

### Insect traction experiments

Insect walking frictional forces were measured on a freshly unrolled (stage 3) and an adult (stage 4) *S. calyptrata* leaf and were compared with the forces on a clean glass slide. The glass slide was cleaned with acetone followed by isopropyl alcohol before performing the traction experiments. For these experiments, female Colorado potato beetles (*Leptinotarsa decemlineata*, Coleoptera: Chrysomelidae) with hairy tarsal attachment system were used as model insect species. The beetles were collected from an organically farmed potato field in Kirchzarten area near Freiburg, Germany, kept in a terrarium and were fed with potato leaves. The lighting conditions were fixed at a day–night regime of 16L:8D by using a lamp (Osram Lumilux Daylight 860, 58 W). Insect traction experiments were performed with a total of seven beetles as described in Prüm et al. [8] and Surapaneni and co-workers [23]. A highly sensitive force transducer (FORT 25, force range: 0–0.25 N, World Precision Instruments Inc., Sarasota, USA) was used to measure maximum walking frictional forces of insects on the leaf surfaces. The elytra of each beetle was attached to the force transducer using a human hair by using a small drop of molten beeswax. The insects were allowed to walk actively for at least 2 min on the experimental samples and the forces were recorded on a computer. Only data from walking in a straight line with less than a ±2° variation were analysed. Within each measurement, the median of the 15 highest local maxima with a minimum interval of 3 s between neighbouring force peaks was extracted. Supporting Information File 3 shows a potato beetle walking on the leaf surface. The average mass of the beetles was 0.17 g. The experiments were conducted under an average temperature of 23.8 ± 0.4 °C and humidity of 41.5 ± 2.1% RH.

### Statistics

The roughness, traction force and cell size data were analyzed using Kruskal–Wallis test followed by pairwise multiple comparisons using Dunn’s test adjusted with the Bonferroni correction. All statistical tests were performed using *R* software environment version 3.6.1 [48]. The experimental data (the raw data and the code) for this study is publicly available on https://freidok.uni-freiburg.de/data/193850 [49].

## Supporting Information

File 1 Additional figures.

File 2 Orientation of ridges in ridge islands and periphery.
The orientation data was collected from the ridge islands and the periphery of a cell using the directionality plugin of the ImageJ software. The data shows typical variation in the ridge orientation (angle with horizontal) at different growth stages.

File 3 Colorado potato beetle (*Leptinotarsa decemlineata*) walking on a *Schismatoglottis* leaf sample.
