# Supplementary material for: Polarity in cuticular ridge development and insect attachment on leaf surfaces of Schismatoglottis calyptrata (Araceae)
Source: Beilstein J Nanotechnol. 2021 Dec 1;12:1326–38. doi: 10.3762/bjnano.12.98 (PMC8649201; doi:10.3762/bjnano.12.98)
Supplement: File 1 — Additional figures. [file Beilstein_J_Nanotechnol-12-1326-s001.pdf]

BEILSTEIN JOURNAL OF NANOTECHNOLOGY

Supporting Information

for

Polarity in cuticular ridge development and insect attachment on leaf surfaces of *Schismatoglottis calyptrata* (Araceae)

Venkata A. Surapaneni, Tobias Aust, Thomas Speck and Marc Thielen

Beilstein J. Nanotechnol. **2021,** *12,* 1326–1338. doi:10.3762/bjnano.12.98

Additional figures

License and Terms: This is a supporting information file under the terms of the Creative Commons Attribution License (https://creativecommons.org/licenses/by/4.0). Please note that the reuse, redistribution and reproduction in particular requires that the author(s) and source are credited and that individual graphics may be subject to special legal provisions.
The license is subject to the *Beilstein Journal of Nanotechnology* terms and conditions: (https://www.beilstein-journals.org/bjnano/terms)

Leaf size versus leaf age in *Schismatoglottis calyptrate*

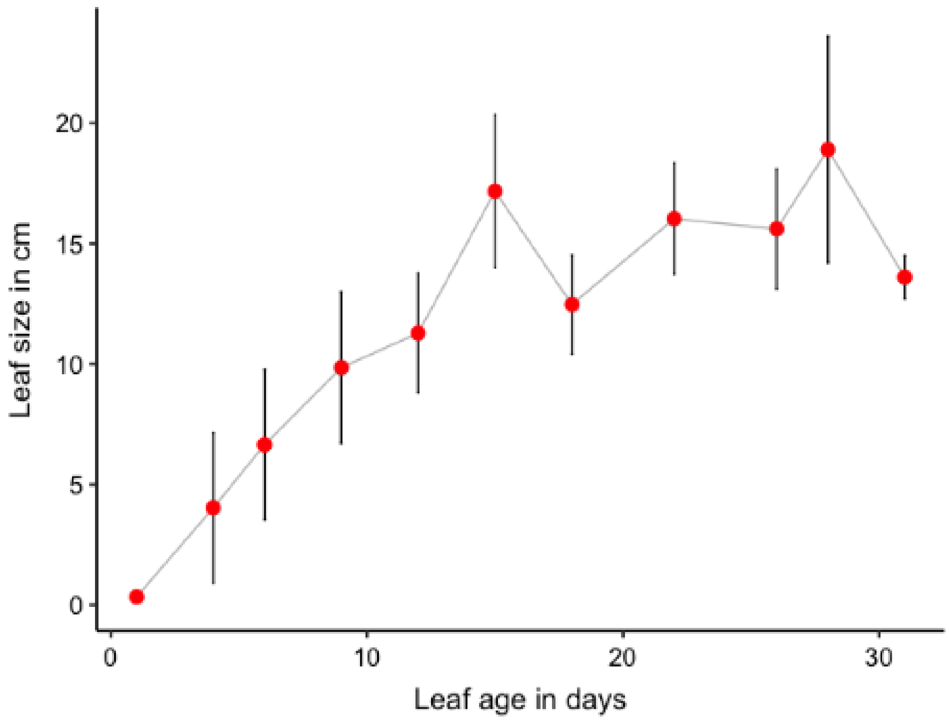

Figure S1: The leaf size was measured from the leaf sheath to leaf tip in rolled leaves in growth stages 1 and 2, and from the base to tip in unrolled leaves in stages 3 and 4. The leaves unrolled between day 12 to 21.

Comparison of characteristic variations in the surface morphology of adaxial *Hevea brasiliensis* and *Schismatoglottis calyptrata* leaves

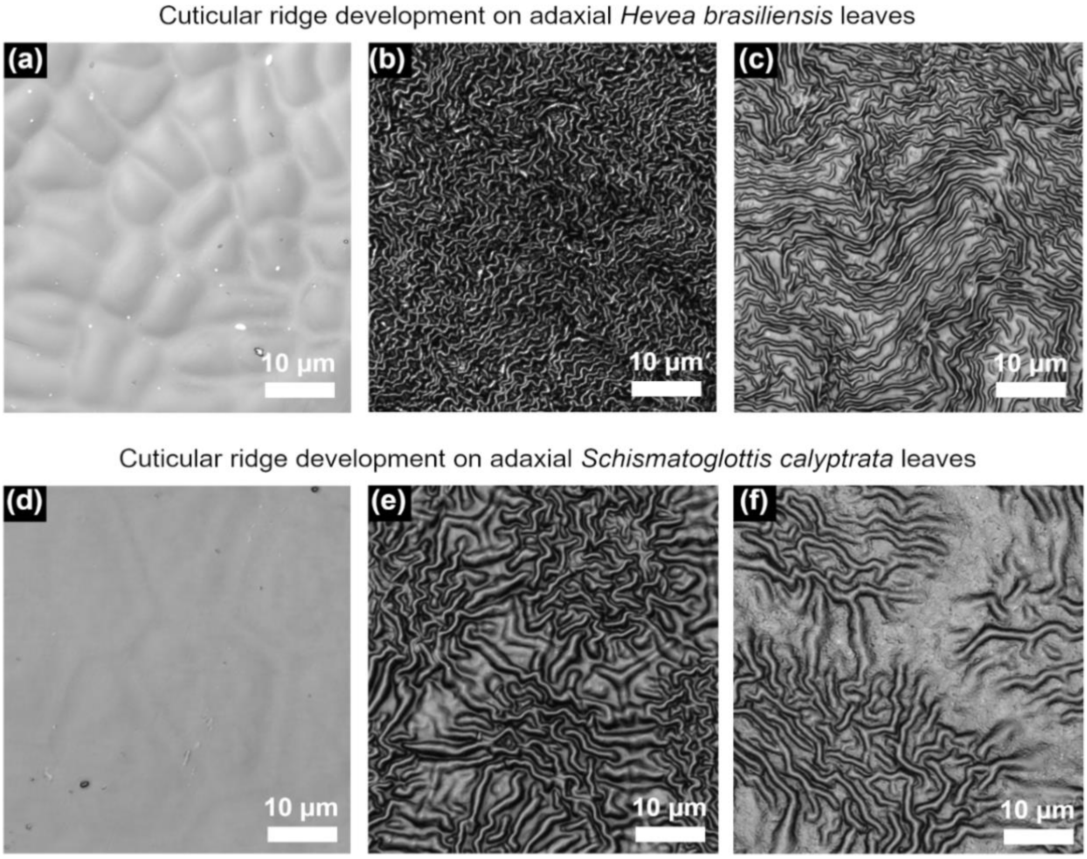

Figure S2: Comparison of characteristic variations in the surface morphology of adaxial *Hevea brasiliensis* (**a-c**), and *Schismatoglottis calyptrata* (**d-f**) leaves. Three distinct levels of surface morphology was observed on the leaves of both species during development – (**a,d**) smooth cells, (**b,e**) high aspect ratio and dense ridges, and (**c,f**) aged ridges with labyrinth-type arrangement in *Hevea brasiliensis* and loosely arranged ridges with smooth anticlinal fields in *Schismatoglottis calyptrata*. Moreover, the ridges on *Schismatoglottis calyptrata* leaves are relatively thicker when compared to those on *Hevea brasiliensis* leaves. **(a-c)** were reproduced from [1] (© 2020 V. A. Surapaneni et al., distributed under the terms of the Creative Commons Attribution 4.0 International License, https://creativecommons.org/licenses/by/4.0). **(d-f)** are identical to Figure 1 **b, c,** and **f** from this paper.

Schematic of division of the *Schismatoglottis calyptrata* leaves

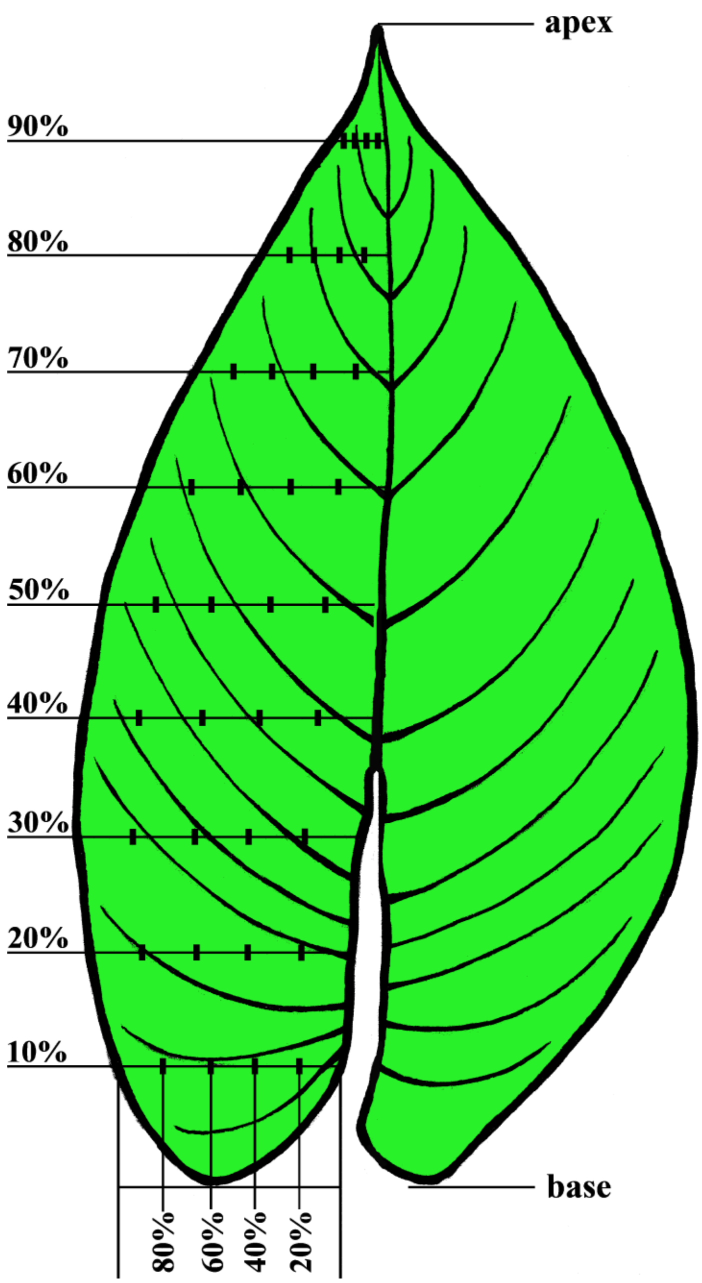

Figure S3: A schematic of division of the *Schismatoglottis calyptrata* leaves to study spatial variations in ridge morphology. A total of 36 spots at the intersections of the divisions on leaf replicas were recorded using confocal laser scanning microscopy.

References

1. Surapaneni, V. A.; Bold, G.; Speck, T.; Thielen, M. *R. Soc. Open Sci.* **2020,** *7,* 201319. doi:10.1098/rsos.201319
