# Supplementary material for: Polarity in cuticular ridge development and insect attachment on leaf surfaces of Schismatoglottis calyptrata (Araceae)
Source: Beilstein J Nanotechnol. 2021 Dec 1;12:1326–38. doi: 10.3762/bjnano.12.98 (PMC8649201; doi:10.3762/bjnano.12.98)
Supplement: File 2 — Orientation of ridges in ridge islands and periphery. The orientation data was collected from the ridge islands and the periphery of a cell using the directionality plugin of the ImageJ software. The data shows typical variation in the ridge orientation (angle with horizontal) at different growth stages. [file Beilstein_J_Nanotechnol-12-1326-s002.pdf]

BEILSTEIN JOURNAL OF NANOTECHNOLOGY

Supporting Information

for

Polarity in cuticular ridge development and insect attachment on leaf surfaces of *Schismatoglottis calyptrata* (Araceae)

Venkata A. Surapaneni, Tobias Aust, Thomas Speck and Marc Thielen

Beilstein J. Nanotechnol. **2021,** *12,* 1326–1338. doi:10.3762/bjnano.12.98

Orientation of ridges in ridge islands and periphery.

The orientation data was collected from the ridge islands and the periphery of a cell using the directionality plugin of the ImageJ software. The data shows typical variation in the ridge orientation (angle with horizontal) at different growth stages

License and Terms: This is a supporting information file under the terms of the Creative Commons Attribution License (https://creativecommons.org/licenses/by/4.0). Please note that the reuse, redistribution and reproduction in particular requires that the author(s) and source are credited and that individual graphics may be subject to special legal provisions.
The license is subject to the *Beilstein Journal of Nanotechnology* terms and conditions: (https://www.beilstein-journals.org/bjnano/terms)

Orientation of ridges in ridge islands and periphery

Detailed description: The orientation data was collected from the ridge islands and the periphery of a cell using the directionality plugin (version 2.3.0) of the ImageJ software. The data shows typical variation in the ridge orientation in a single ridge island and periphery (angle with horizontal – perpendicular to leaf's midrib) at different growth stages in the form of frequency histograms.

Stage 2A

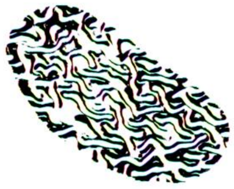

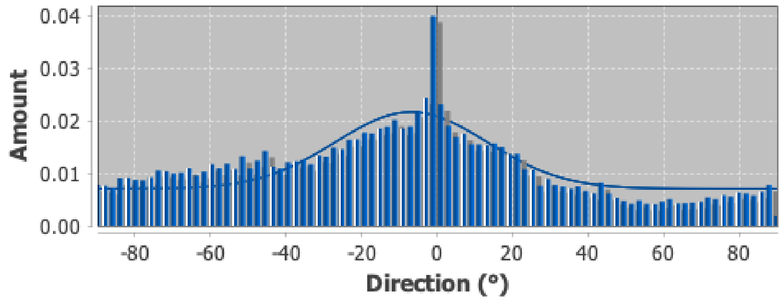

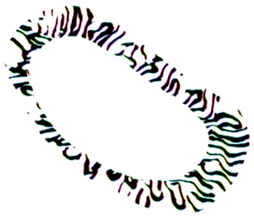

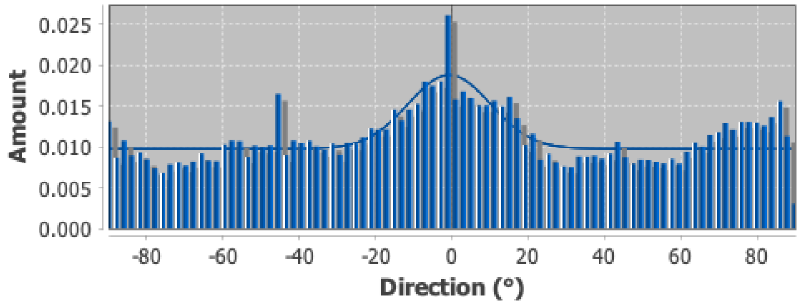

Stage 3

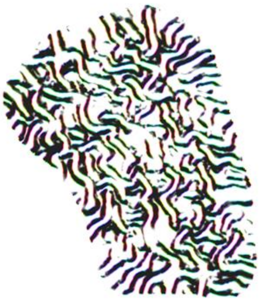

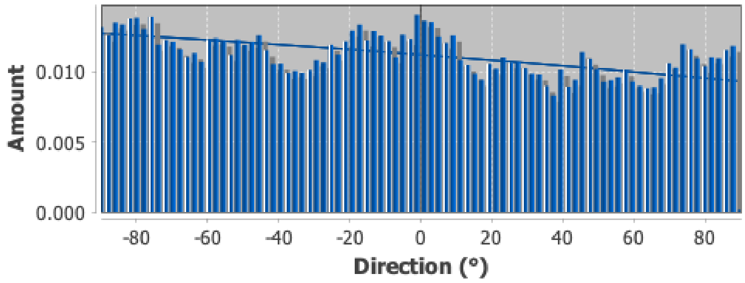
Amount
0.010
0.005
0.000
-80
-60
-40
-20
0
20
40
60
80
Direction (°)

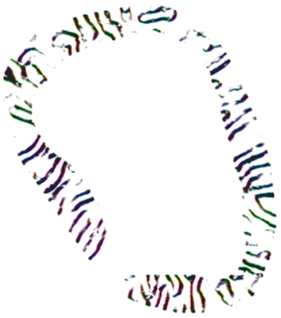

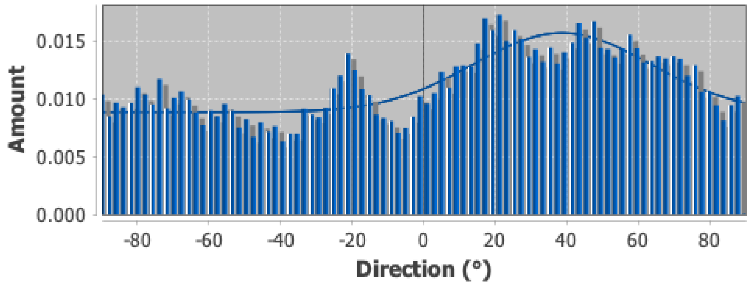
Amount
0.015
0.010
0.005
0.000
-80
-60
-40
-20
0
20
40
60
80
Direction (°)

Stage 4

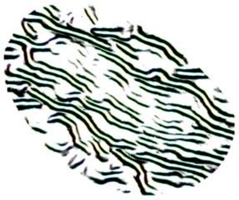	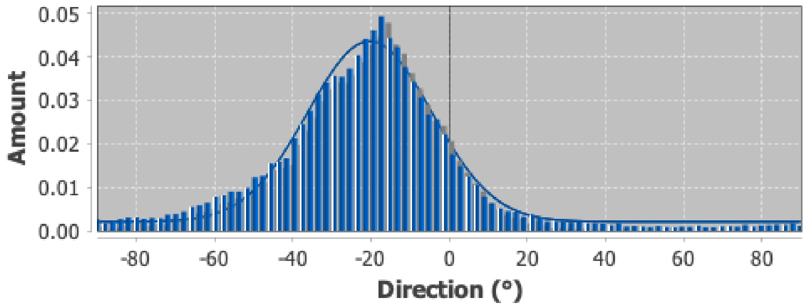Amount<br>0.05<br>0.04<br>0.03<br>0.02<br>0.01<br>0.00<br>-80 -60 -40 -20 0 20 40 60 80<br>Direction (°)
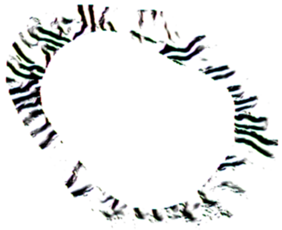	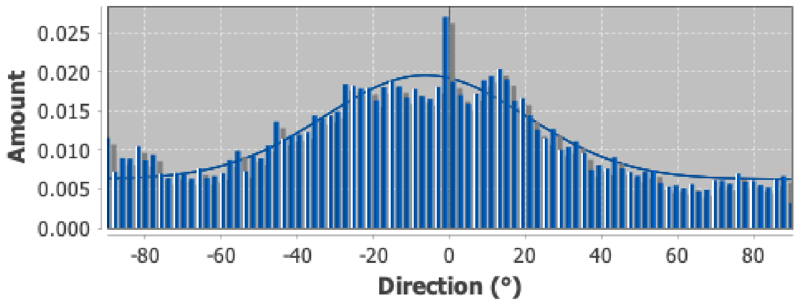Amount<br>0.025<br>0.020<br>0.015<br>0.010<br>0.005<br>0.000<br>-80 -60 -40 -20 0 20 40 60 80<br>Direction (°)
